# Tubal Pregnancy Associated with Endometrial Carcinoma after In Vitro Fertilization Attempts

**DOI:** 10.1155/2014/481380

**Published:** 2014-12-28

**Authors:** Yesim Bayoglu Tekin, Emine Seda Guvendag Guven, Ibrahim Sehitoglu, Suleyman Guven

**Affiliations:** ^1^Recep Tayyip Erdogan Universitesi, Tıp Fakultesi Dekanlığı, Islampaşa Mahallesi, Merkez, 53020 Rize, Turkey; ^2^Department of Gynecology and Obstetrics, School of Medicine, Recep Tayyip Erdogan University, 53020 Rize, Turkey; ^3^Department of Pathology, School of Medicine, Recep Tayyip Erdogan University, 53020 Rize, Turkey; ^4^Department of Gynecology and Obstetrics, School of Medicine, Black Sea Technical University, 61200 Trabzon, Turkey

## Abstract

Endometrial carcinoma is rarely seen during reproductive ages and commonly related to infertility, polycystic ovarian syndrome (PCOS), and obesity. Pregnancy associated endometrial carcinoma is even rarer and this is the second case reported in the literature concerning tubal pregnancy associated endometrial carcinoma. We present a case of a 36-year-old woman with a history of PCOS, infertility, and several attempts of ovulation induction and in vitro fertilization, who was diagnosed with tubal pregnancy and a well differentiated endometrial carcinoma. We also review the literature about pregnancy associated endometrial carcinoma in the first trimester.

## 1. Introduction

Endometrial cancer is the most common cancer of the female reproductive tract, which occurs very rarely in pregnancy [1]. Most of the reported cases occurred during the first trimester. There is only one case report of endometrial cancer cooccurring with ectopic tubal pregnancy [2]. Endometrial cancer rarely occurs before the age of 40. Endometrial cancers fall into two groups according to their developmental patterns. Type I endometrial cancer, which is hyperplastic in origin, is associated with a younger age at menarche and late age at menopause and nulliparity. Type 2 endometrial cancer is related to atrophy. Standard treatment for early stage endometrial cancer is surgery including hysterectomy and bilateral salpingooophorectomy. However, conservative treatment is preferred at the reproductive ages for fertility sparing. The present report describes a case of endometrial cancer coexisting with tubal ectopic pregnancy that had a history of infertility, chronic anovulation, and several failed attempts of infertility treatments, in addition to a review of the literature.

## 2. Case Report

A 36-year-old woman with gravida 0 had a history of infertility and clinical diagnosis of polycystic ovarian disease since the age of 22. Her menstrual cycles were irregular with oligomenorrhea and menometrorrhagia.

She had received 3 cycles of clomiphene citrate and 2 cycles of ovulation induction (OI) and artificial insemination before the in vitro fertilization (IVF) attempts. She was administered the luteal long gonadotropin releasing hormone (GnRH) protocol in the first and second attempts and the GnRH antagonist protocol in the third attempt. In the last IVF treatment cycle, 5 oocytes were yielded and two good quality embryos were transferred on day 3.

**Figure 1:**
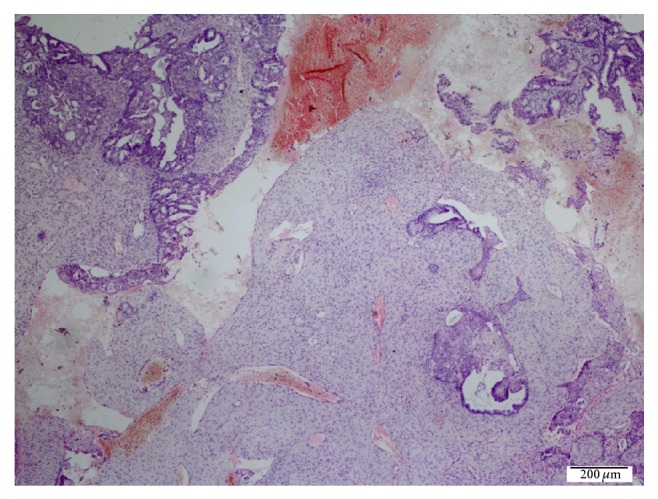
The nests of well differentiated endometrial carcinoma in the decidual reaction (HE ×40).

The patient was admitted to our clinic due to abnormal vaginal bleeding, whose last menstrual period was 5 weeks earlier following the third IVF attempt. She was 150 cm in height and 64 kg in weight with a body mass index of 28.4 kg/m^2^ who was found to be normotensive on physical examination. She had no remarkable abdominal or pelvic pain and no tenderness on abdominal examination. Vaginal spotting was observed in speculum examination. Complete blood cell count, urinalysis, and coagulation values were within normal limits. The blood chorionic gonadotropin (*β*hCG) level was 2226 mIU/mL. Endovaginal ultrasonography revealed irregular lining of endometrium with 21 mm thickness, enlarged diameters of bilateral ovaries with multiple corpus luteomas, and minimal free fluid at the Douglas pouch. The patient was monitored for *β*hCG elevation. Two days after the first evaluation of the patient, the endometrial thickness slightly increased giving the pseudogestational sac appearance. Imaging revealed increased free fluid at 33 mm depth and a suspicious mass in the right adnexal region adherent to the ovary which was giving the impression of ectopic pregnancy. The blood *β*hCG level was elevated to 4406 mIU/mL. A dilatation and curettage was performed after the administration of methotrexate (50 mg/m^2^) for excluding the heterotopic pregnancy. Pathologic examination revealed well differentiated endometrial carcinoma with decidual reaction and no chorionic villi (Figure 1).

The blood *β*hCG levels were slightly decreased and became undetectable after 4 weeks following the administration of methotrexate. Medroxyprogesterone acetate therapy and repeat curettages were applied for the treatment of the endometrial carcinoma. A year later, a new IVF attempt was launched.

## 3. Discussion

Endometrial cancer is usually seen in advanced ages. Risk factors include diabetes, hypertension, and a history of chronic exposure to unopposed estrogen in obesity, infertility, and PCOS. The prevalence of endometrial cancer in the reproductive age is 5% of all endometrial carcinomas [3]. PCOS, infertility, and relevant treatments are among the causes of endometrial cancers in early ages. Pregnancy associated endometrial cancer is a very rare condition and this is the second case of tubal pregnancy associated endometrial cancer in the literature.

There were twenty cases of pregnancy associated endometrial carcinoma in the first trimester in the literature (Table 1) [4–18], only one of which was tubal ectopic pregnancy coexisting with endometrial carcinoma [2] and the others were with intrauterine pregnancies. The mean age and gravidity were, respectively, 35.7 ± 6.7 (21–45) and 1.7 ± 2.3 (1–10). 40% of the patients had a history of infertility. Most of the patients presented with abnormal vaginal bleeding and diagnosis was made frequently during curettage for elective or spontaneous abortion [1]. The mean gestational age was 8.3 ± 2.6 (3–13) during diagnosis. The majority of the patients were in the early stages without myometrial invasion and distant metastasis similar to the other endometrial carcinomas that were seen in the fertile ages [19]. The majority of the patients (70%) underwent radical therapy in the form of hysterectomy and bilateral salpingooophorectomy. Fertility sparing therapy by administration of progesterone and repeat follow-up curettages were applied to only 25% of the women [20].

Our case had some risk factors for the development of endometrial cancer. Firstly, she had a history of PCOS and oligoanovulatory cycles. It is known that PCOS patients are at risk of endometrial cancer due to prolonged anovulatory cycles and exposure to unopposed estrogen. It is likely that there is an elevated coactivator activity in the endometrium of anovulatory woman with PCOS, predisposing to estrogen-induced hyperplasia and cancer [21].

The second risk factor was infertility treatment with several attempts of controlled ovarian stimulation (COH) and IVF. It was previously reported that clomiphene citrate increased the endometrial cancer rate [22]. Moreover infertility treatments by ovulation induction or COH cause a hyperestrogenic milieu by the supraphysiological gonadotropin levels that could provide the maintenance and progression of endometrial carcinoma [18]. IVF treatment is found to be associated with increased risk of endometrial cancer [20].

Coexistence of endometrial cancer and pregnancy in the first trimester should be suggestive of preexisting endometrial neoplasia. The elevated progesterone levels in pregnancy are unfavorable for the development and progression of endometrial cancer. Pregnancy associated endometrial carcinoma develops from an endometrial focus with impaired receptivity that is sensitive to estrogen and resistant to progesterone [13]. Thus the refractory region progresses to carcinoma and the rest of the endometrium continues to respond to estrogen and progesterone stimuli.

Moreover, endometrial cancer causes the impairment of endometrial receptivity and implantation defect. Successful implantation requires cross talk between the developing embryo and receptive endometrium in a restricted time period as defined by the implantation window [23]. Regulation of progesterone hormone receptors plays a critical role in implantation of the embryo and decidualization of the endometrium [24]. The possible cause of the implantation of the embryo to the tubal epithelium in this case could be the endometrial like cyclic changes in the tubal epithelium allowing the attachment of the embryo that failed to live in the endometrium with impaired receptivity [22]. Furthermore GnRH antagonist treatments could be the other cause of the faulty implantation due to adverse effect on the endometrial receptivity [25].

In conclusion, pregnancy associated endometrial carcinoma is a rare event; however it is closely associated with infertility. Therefore, PCOS patients exposed to prolonged infertility therapies should be evaluated for endometrial pathologies secondary to infertility treatments if clinical scenario suggests it.

## Figures and Tables

**Table 1:** List of the cases with pregnancy associated with endometrial carcinoma in the first trimester.

Reference	Age	Symptom	Parity	Gestational week	Diagnosis	Histopathology	Treatment
Schmumann et al. [4]	43	Bleeding	10	10–12	D & C	G1	TAH + BSO

Karlen et al. [5]	21	Bleeding	2	6–8	D & C	G1 with squamous differentiation	TAH

Sandstrom et al. [6]	37	Spotting	0	10	D & C	G1 with squamous differentiation	TAH + BSO

Zirkin et al. [7]	42	Bleeding	4	1st tr.	D & C	G1 superficial myometrial invasion	TAH + BSO

Suzuki et al. [8]	30	Bleeding	1	7	D & C	G2 > 50% myometrial invasion	TAH + BSO, RT

Pulitzer et al. [9]	33	Ectopic pregnancy?	0	1st tr.	Laparoscopy for adnexal mass	G1 endometrial Ca+ incidental bilateral ovarian CA	TAH + BSO

Carinelli et al. [10]	40	Amenorrhea	2	1st tr.	D & C	Focal G3	Repeat D & C

Hoffman et al. [11]	35	None	0	8-9	D & C	Focal G2, synchronous ovary G2	TAH + BSO,

Orlov and Grigor'ev [12]	42	Bleeding	2	9	D & C	G1	TAH + BSO

Schneller and Nicastri [13]	26	Amenorrhea	0	3	D & C	G1	Repeat D & C

Kovács and Cserni [14]	35	Bleeding	1	7	D & C	G1-G2 superficial myometrial invasion	TAH + BSO, RT

Stead and Behnam [2]	28	Spotting	2	4	D & C	G2	TAH + BSO

Schammel et al. [15]	38	Infertility	0	9	D & C	G1	Repeat D & C
	41	Bleeding	0	13	D & C	G1	TAH + BSO
	29	None	2	9-10	D & C	G1	NA
	34	Bleeding	0	13	D & C	G1	Progesterone + D & C

Ayhan et al. [16]	44	Bleeding	2	5	D & C	G1	TAH + BSO

Vaccarello et al. [17]	35	Bleeding	0	7	D & C	Focal G1	TAH + BSO

Yael et al. [1]	39	None	4	12	D & C	G1	Progesterone + D & C

Akil et al. [18]	45	Bleeding	3	8	D & C	G1	TAH + BSO

D & C: dilatation and curettage, G: grade, TAH + BSO: total abdominal hysterectomy and bilateral salpingooophorectomy, RT: radiotherapy, tr.: trimester, and NA: not available.

^*^The case of tubal pregnancy associated with endometrial carcinoma.
